# Supplementary material for: Loss of heterozygosity of essential genes represents a widespread class of potential cancer vulnerabilities
Source: Nat Commun. 2020 May 20;11:2517. doi: 10.1038/s41467-020-16399-y (PMC7239950; doi:10.1038/s41467-020-16399-y)
Supplement: Supplementary file 1 — Supplementary Information [file 41467_2020_16399_MOESM1_ESM.pdf]

Supplementary Information

Loss of heterozygosity of essential genes represents
a widespread class of cancer vulnerabilities

Nichols et al.

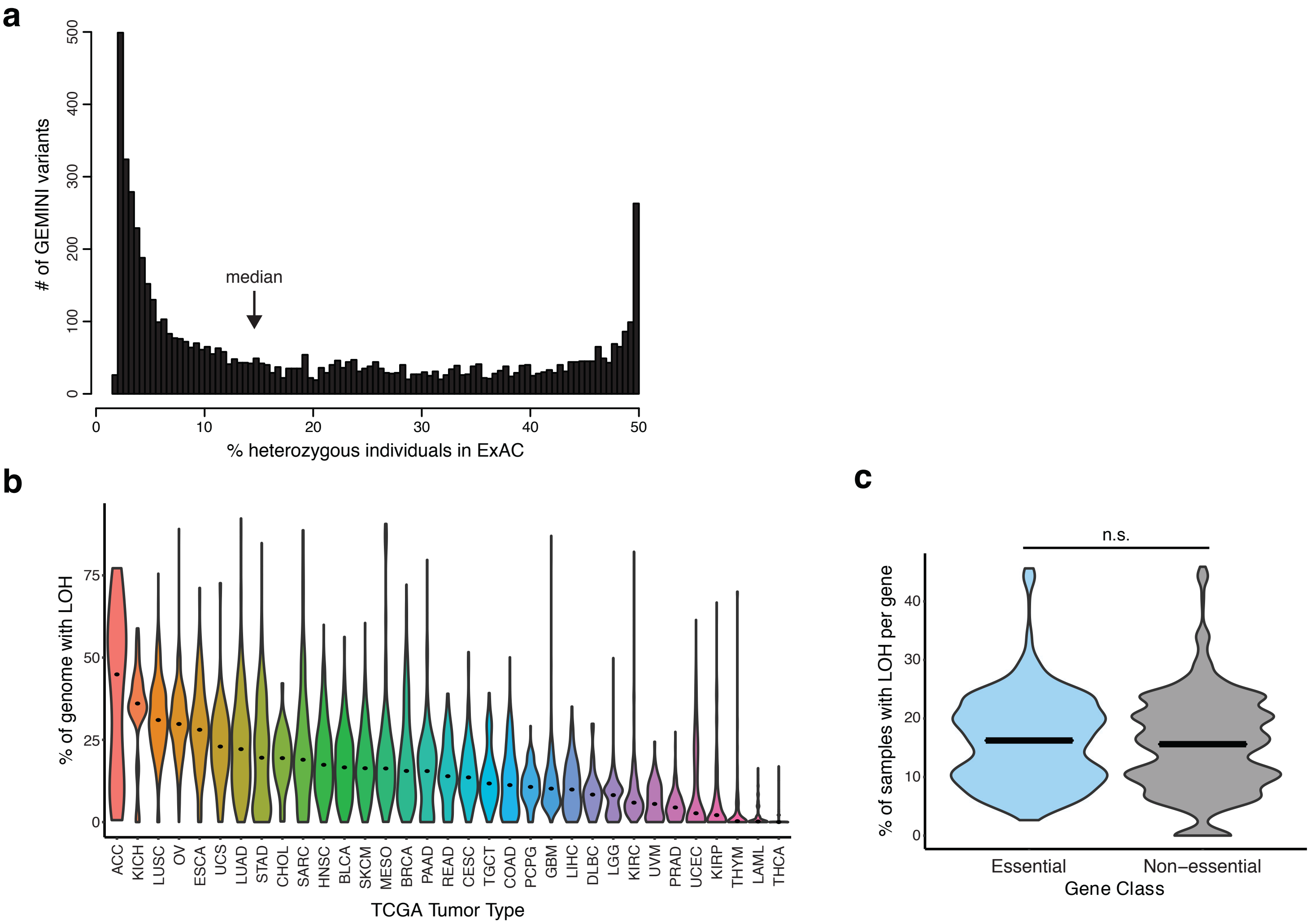

Supplementary Figure 1: Additional genomic characterization of LOH of essential genes.

a. Number of GEMINI variants (vertical axis) plotted against the fraction of individuals that are heterozygous for each variant in the ExAC cohort (horizontal axis). Bin width = 0.5%. **b.** Violin plot of the percent of genome affected by LOH across 33 TCGA tumor types. Tumor types are indicated by TCGA abbreviations (see https://gdc.cancer.gov/resources-tcga-users/tcga-code-tables/tcga-study-abbreviations). Plot width represents relative sample density and dots indicate median values. **c.** Violin plot demonstrating the rate of LOH for essential (blue) and non-essential genes (grey). Essential genes do not have a significantly higher rate of LOH (One-tailed Student's t-test, p=1 [n.s. = not significant]). Intersecting lines indicate median values.

a

PRIM1^{rs2277339}	
position	chr12:57146069
variant	T/G
consequence	missense variant (D5A)
minor allele frequency	0.177
predicted frequency of heterozygosity	0.291
pan-cancer LOH frequency	0.089
theoretical US patients per year	22,470

b

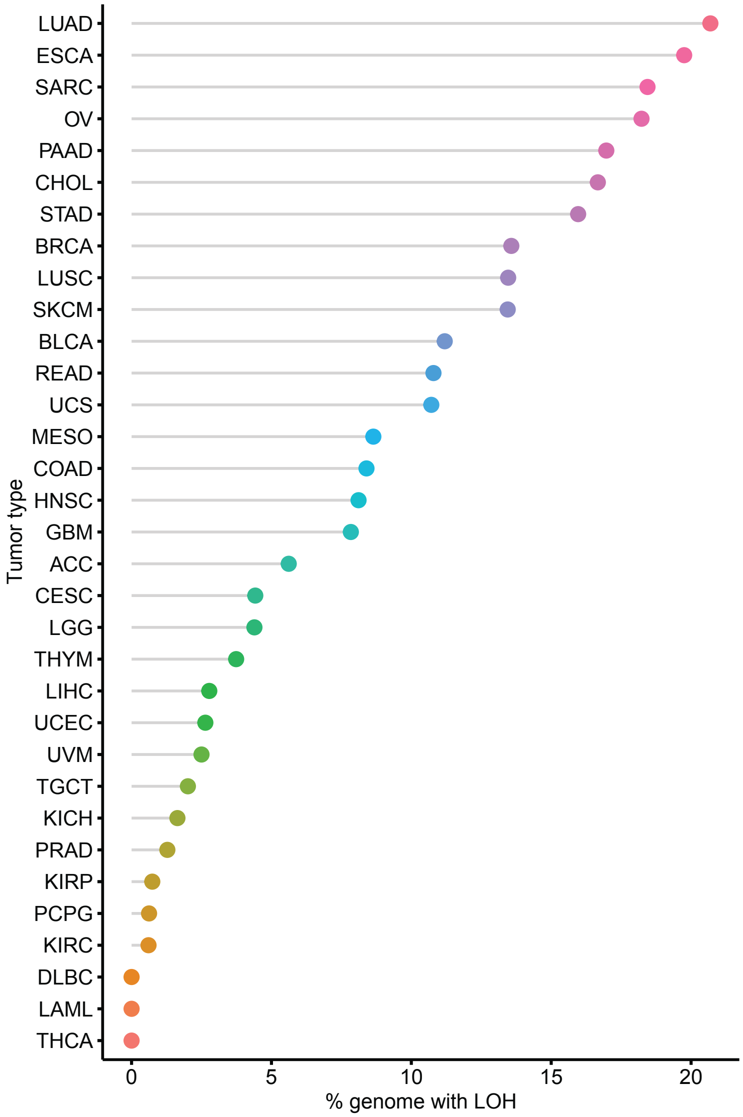

Supplementary Figure 2: Additional *PRIM1* statistics and proliferation data.

a. Table of *PRIM1*^{rs2277339} SNP statistics, including calculation of theoretical number of treatable patients in the US per year (see Methods). **b.** Cleveland dot plot of the rate of *PRIM1* LOH across 33 TCGA tumor types. Tumor types are indicated by TCGA abbreviations (see https://gdc.cancer.gov/resources-tcga-users/tcga-code-tables/tcga-study-abbreviations).

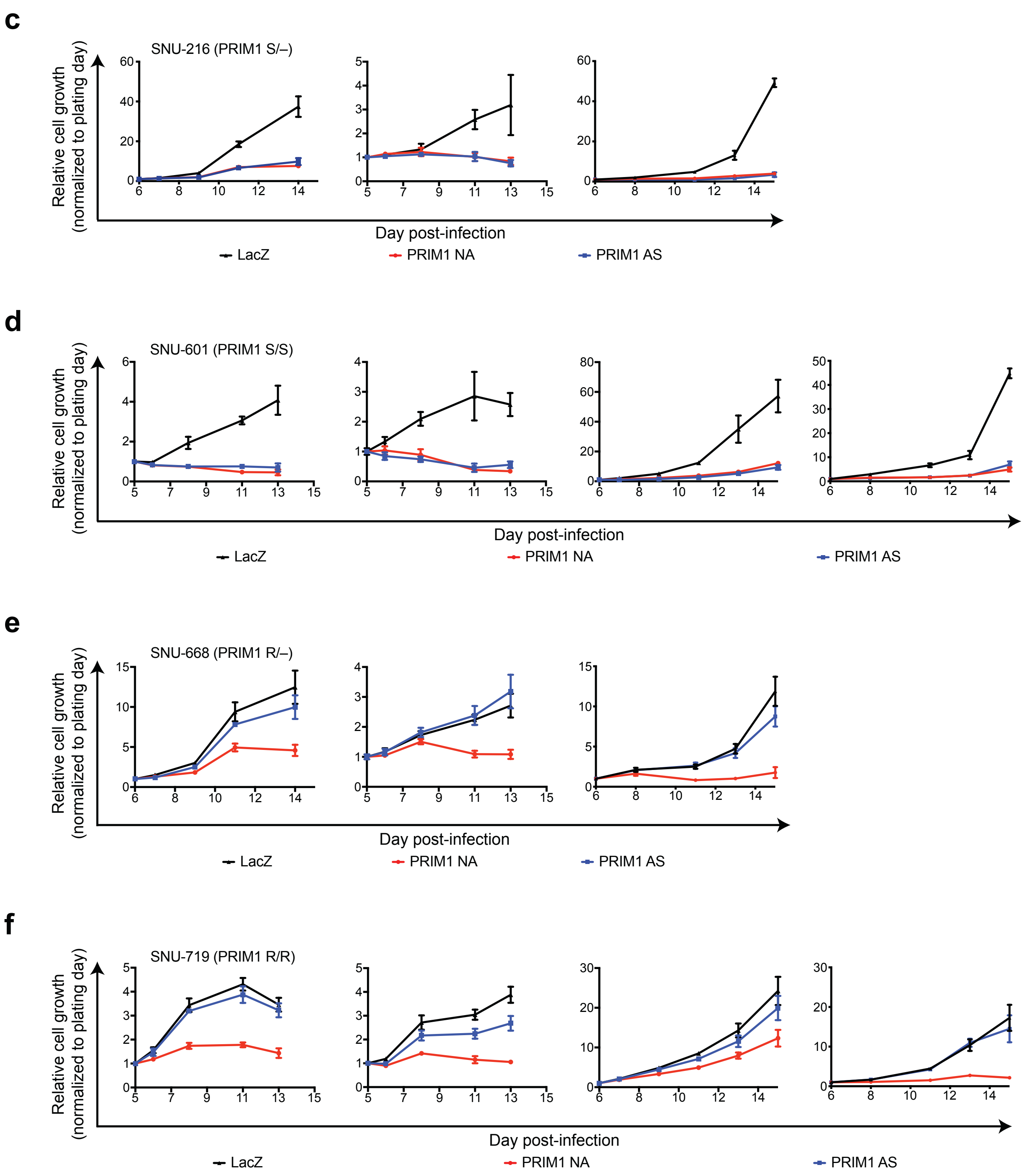

Supplementary Figure 2: Additional *PRIM1* statistics and proliferation data.
c.–f. Growth of indicated patient-derived cell lines expressing LacZ (black), PRIM1 NA (red), or PRIM1 AS (blue) sgRNA as measured by CellTiter-Glo luminescence, relative to day of assay plating. n = 5 technical replicates. Data are presented as mean values +/- s.d. Source data provided as a Source Data file.

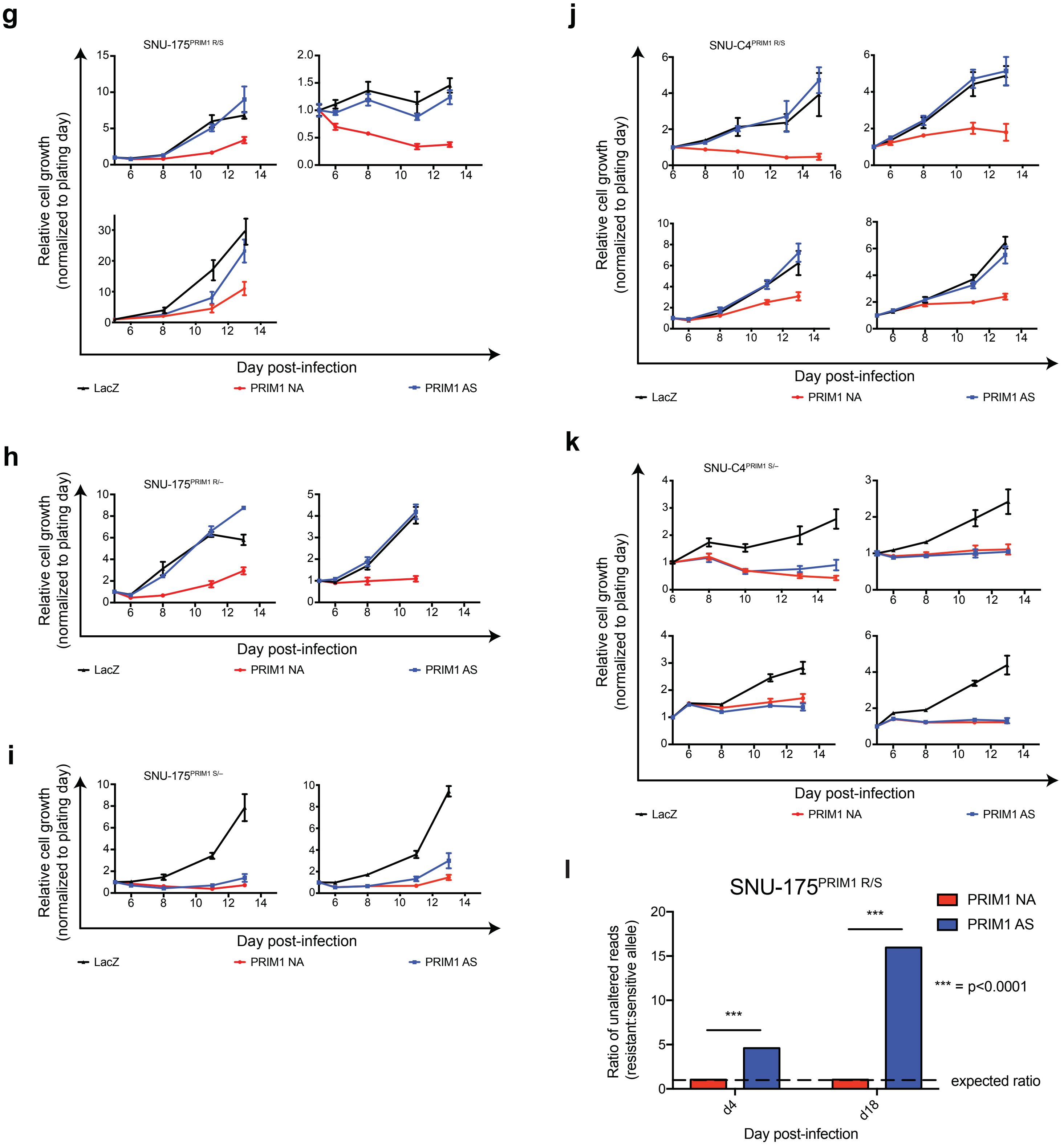

Supplementary Figure 2: Additional *PRIM1* statistics and proliferation data.

g.–k. Growth of indicated isogenic cell lines expressing LacZ (black), PRIM1 NA (red), or PRIM1 AS (blue) sgRNA as measured by CellTiter-Glo luminescence, relative to day of assay plating. n = 5 technical replicates. Data are presented as mean values +/- s.d. **l.** Ratio of unaltered resistant to unaltered sensitive alleles of *PRIM1*^{rs2277339} in *PRIM1* heterozygous cells (PRIM1$^{R/S}$) at day 4 and day 18 post-infection with NA or AS sgRNA as assessed by deep sequencing. n = 1 biologically independent experiment. d4: PRIM1 NA—17,008 R:16,296 S read pairs; PRIM1 AS—30,152 R:6544 S read pairs. d18: PRIM1 NA—20,950 R:20,081 S read pairs; PRIM1 AS—30,209 R:1906 S read pairs. Dashed line indicates expected ratio of unaltered R:S alleles if an sgRNA targets either allele with equal frequency. Chi-square with Yates correction, ***p<0.0001. Source data provided as a Source Data file.

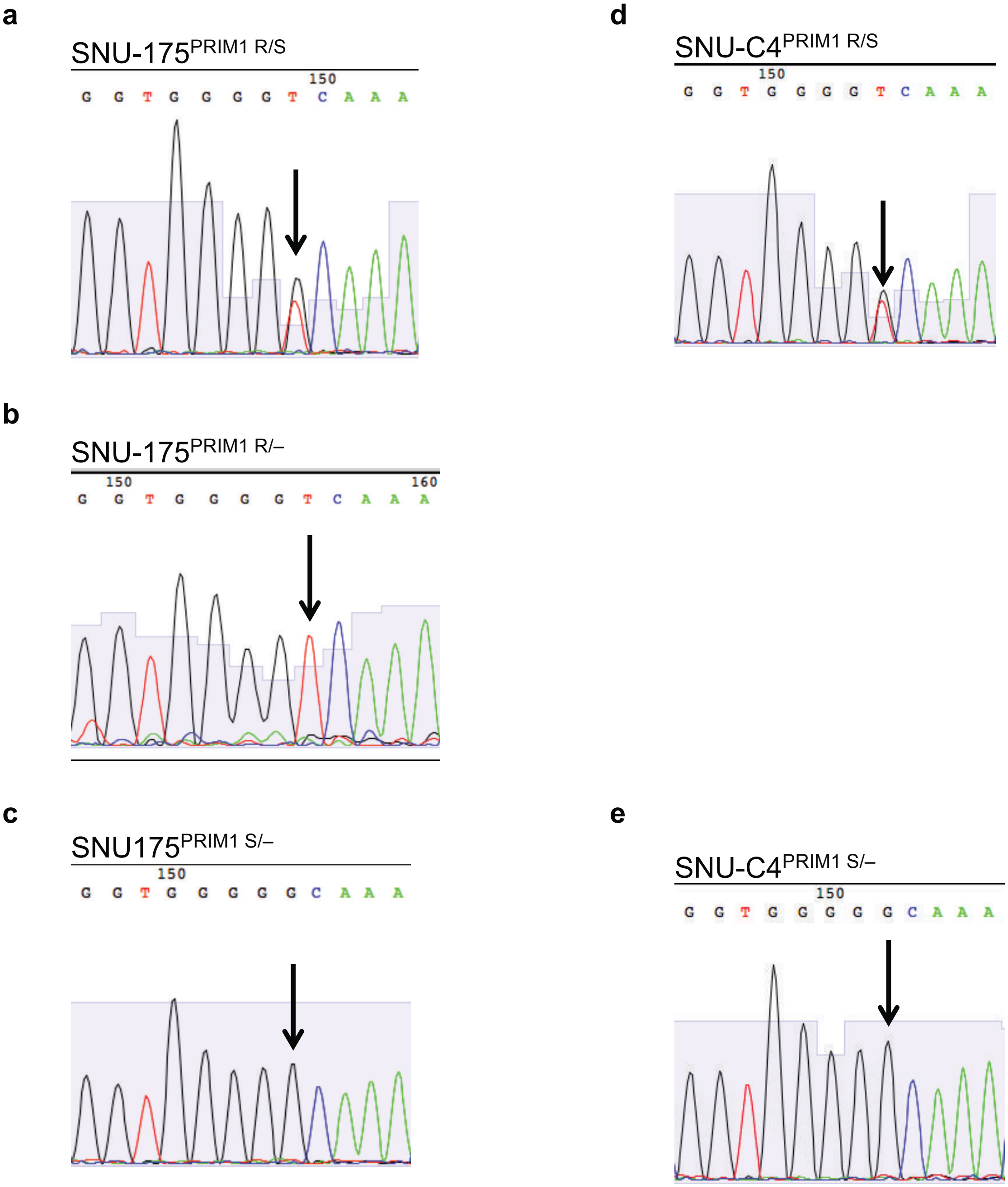

Supplementary Figure 3: Genotypic characterization of isogenic cell lines.
a.–e. Genotyping of *PRIM1*^{rs2277339} locus in indicated isogenic cell lines by Sanger sequencing. Sequencing peaks representing SNP loci indicated by black arrow.

resistant (R) allele
sensitive (S) allele
editing sgRNA blocking mutation
synonymous mutation introducing MnlI cut site
insertion
deletion

f

DV-90^EXOSC8 R/R^

Reference
```
AGCTGCAGAGTGTTTCTTTCAGTTCCTAATGTGGATCTACCACCCCTGTGTTCATCGAGA
```

Edited R allele, 88,509 read pairs
```
AGCTGCAGAGTGTTTCTTTCAGTTCCTAATGTGGATCTACCTCATCTGTGTtcatcgaga
***************************************** *  **************
```

g

DV-90^EXOSC8 R/Δ^

Reference
```
AGCTGCAGAGTGTTTCTTTCAGTTCCTAATGTGGATCTACCACCCCTGTG-TTCATCGAG
```

Edited R allele, 44,454 read pairs
```
AGCTGCAGAGTGTTTCTTTCAGTTCCTAATGTGGATCTACCTCATCTGTG-Ttcatcgag
```

Disrupted S allele, 42,169 read pairs
```
AGCTGCAGAGTGTTTCTTTCAGTTCCTAATGTGGATCTACCACCCCTGTGTTtcatcgag
***************************************** *  ***** *********
```

h

DV-90^EXOSC8 S/S^

Reference
```
AGCTGCAGAGTGTTTCTTTCAGTTCCTAATGTGGATCTACCACCCCTGTGTTCATCGAGA
```

Edited S allele, 54,799 read pairs
```
AGCTGCAGAGTGTTTCTTTCAGTTCCTAATGTGGATCTACCTCCGCTGTGTtcatcgaga
***************************************** ** ***************
```

i

DV-90^EXOSC8 S/Δ^

Reference
```
AGCTGCAGAGTGTTTCTTTCAGTTCCTAATGTGGATCTACCACCCCTGTGTTCATCGAGA
```

Disrupted S allele, 22,831 read pairs
```
AGCTGCAGAGTGTTTCTTTCAGTTCCTAATGTGGATCTACCACCCCTGTG-TCAtcgaga
```

Edited S allele, 23,409 read pairs
```
AGCTGCAGAGTGTTTCTTTCAGTTCCTAATGTGGATCTACCTCCGCTGTGTTCAtcgaga
***************************************** ** ***** *********
```

Supplementary Figure 3: Genotypic characterization of isogenic cell lines.
f.–i. Genotyping of *EXOSC8*^rs117135638^ locus in indicated isogenic cell lines by next-generation sequencing. Relevant sequence features indicated by font or highlighting color. Off-target reads and species representing less than 0.05% of total read pairs removed for clarity.

a

$EXOSC8^{rs117135638}$	
position	13:37580078
variant	C/A
consequence	missense variant (P87H)
minor allele frequency	0.011
predicted frequency of heterozygosity	0.021
pan-cancer LOH frequency	0.291
theoretical US patients per year	5307

b

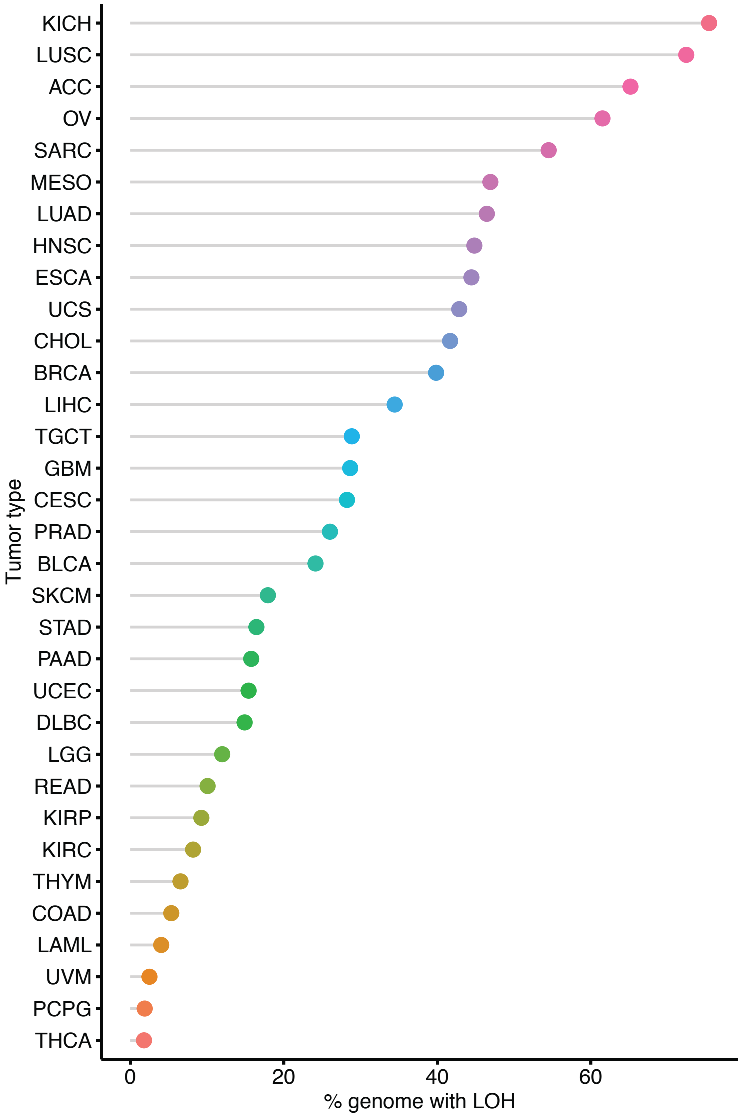

Supplementary Figure 4: Additional *EXOSC8* statistics and proliferation data.

a. Table of $EXOSC8^{rs117135638}$ SNP statistics, including calculation of theoretical number of treatable patients in the US per year (see Methods). **b.** Cleveland dot plot of the rate of *EXOSC8* LOH across 33 TCGA tumor types. Tumor types are indicated by TCGA abbreviations (see https://gdc.cancer.gov/resources-tcga-users/tcga-code-tables/tcga-study-abbreviations).

c

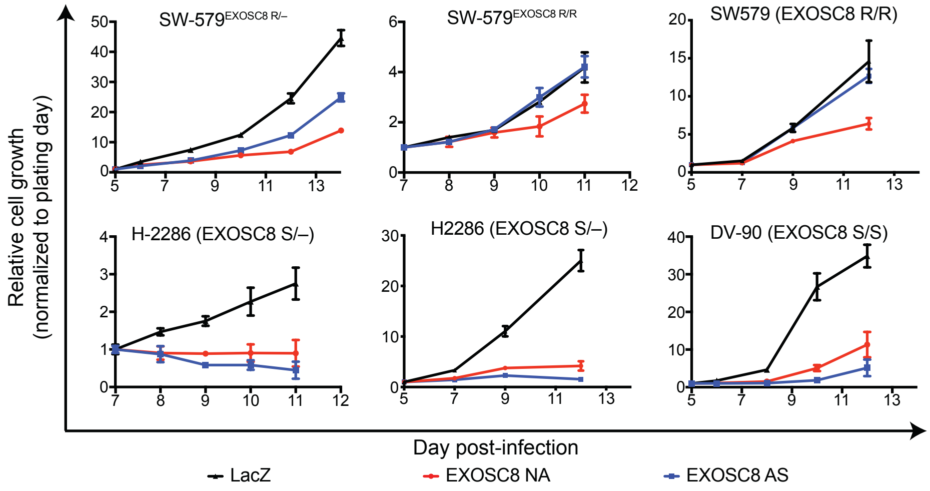

d

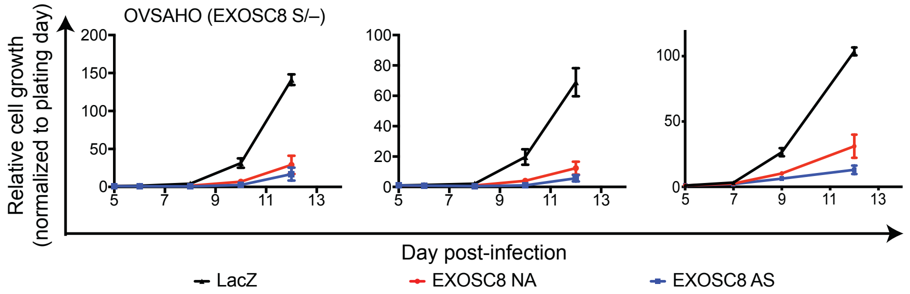

e

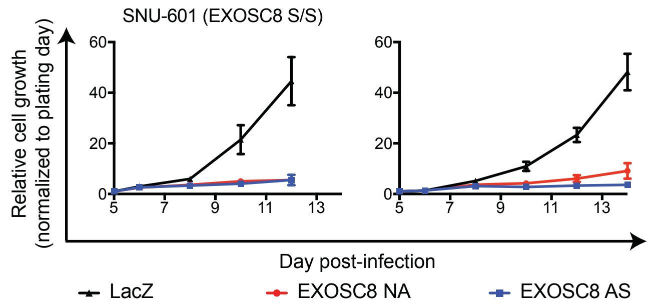

f

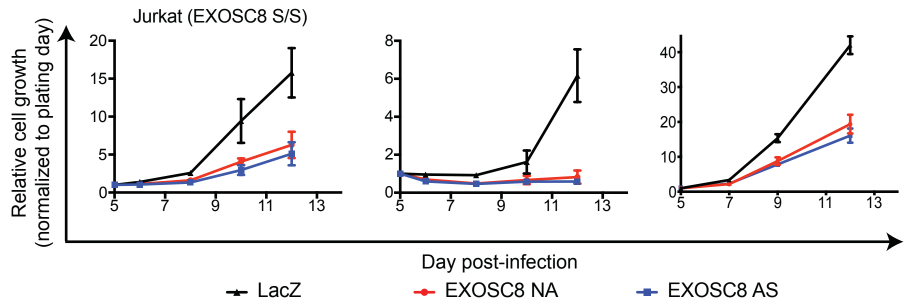

Supplementary Figure 4: Additional *EXOSC8* statistics and proliferation data.
c.–f. Growth of indicated patient-derived cell lines expressing LacZ (black), EXOSC8 NA (red), or PRIM1 AS (blue) sgRNA as measured by CellTiter-Glo luminescence, relative to day of assay plating. n = 5 technical replicates. Data are presented as mean values +/- s.d. Source data provided as a Source Data file.

g

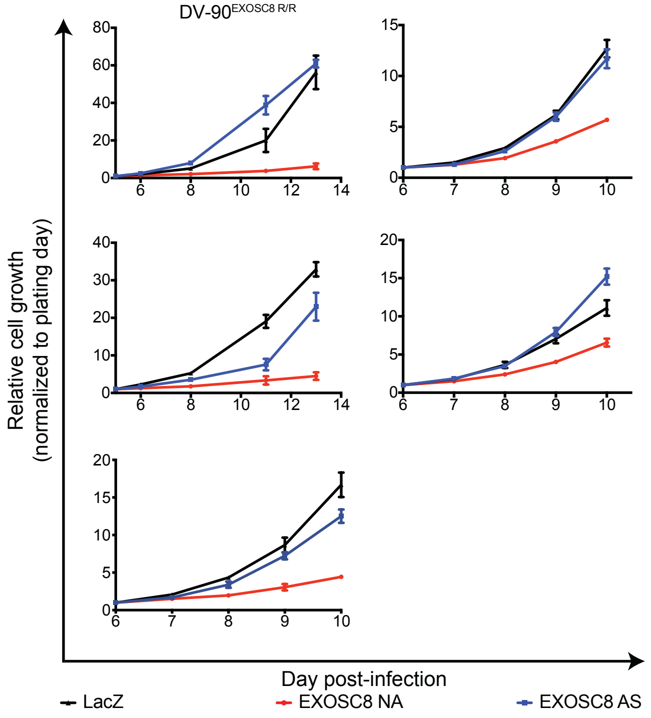

i

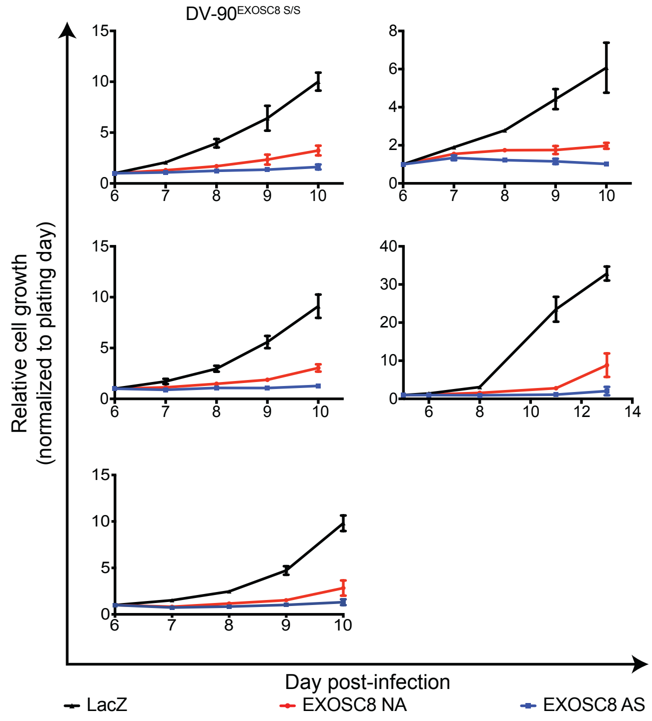

h

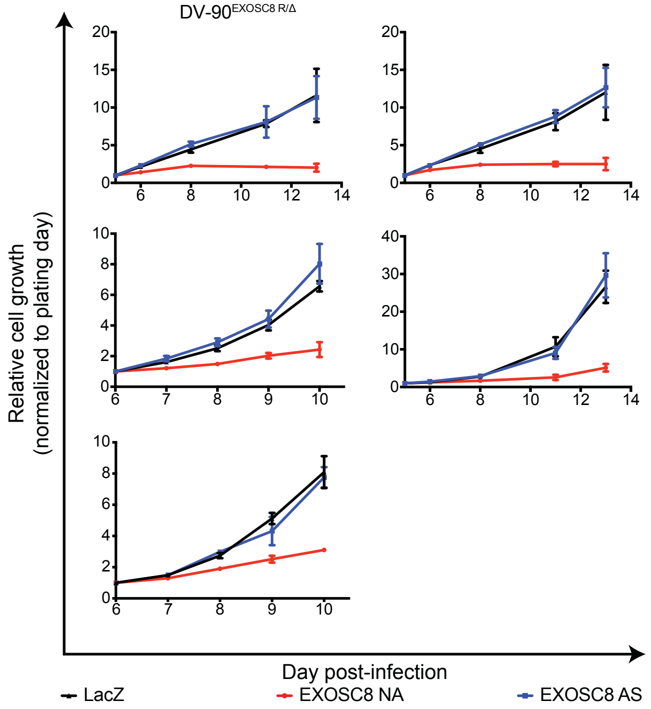

j

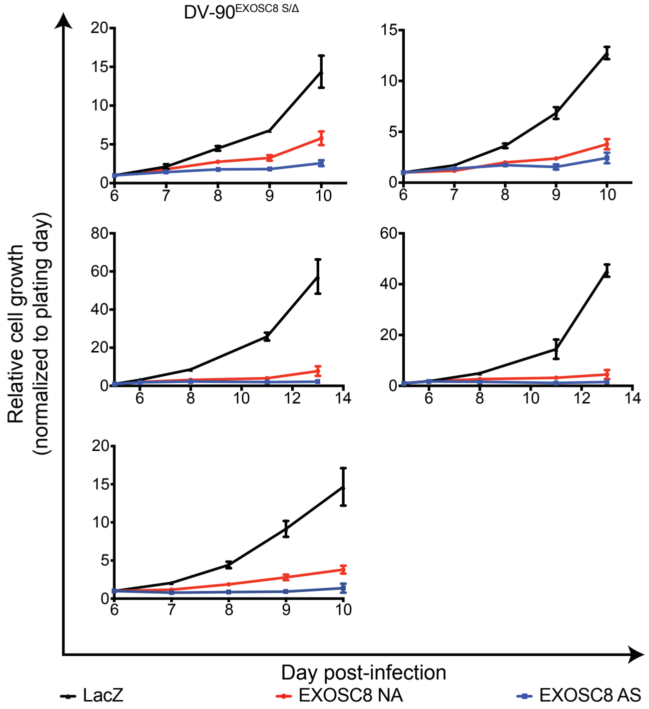

Supplementary Figure 4: Additional *EXOSC8* statistics and proliferation data.
g.–j. Growth of indicated isogenic cell lines expressing LacZ (black), EXOSC8 NA (red), or EXOSC8 AS (blue) sgRNA as measured by CellTiter-Glo luminescence, relative to day of assay plating. n = 5 technical replicates. Data are presented as mean values +/- s.d. Source data provided as a Source Data file.

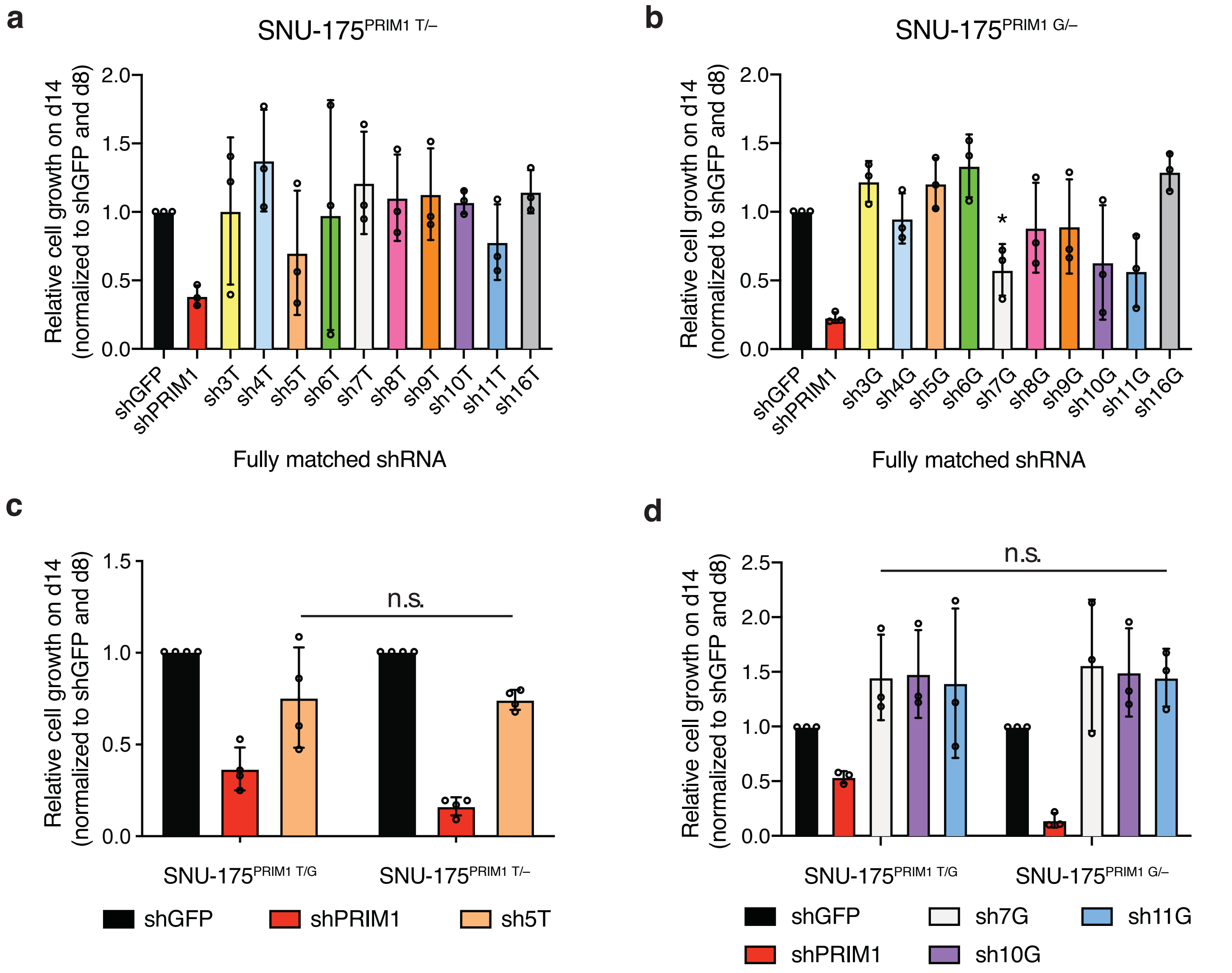

Supplementary Figure 5: Growth of cells expressing putative *PRIM1*rs2277339-specific shRNAs.
a. Growth of cells hemizygous for the major *PRIM1*rs2277339 allele (SNU-175PRIM1 T/–) expressing shGFP, positive control shPRIM1, or indicated putative allele-specific shRNAs targeting the T allele of *PRIM1*rs2277339, as measured by CellTiter-Glo luminescence, relative to day of assay plating. n = 3 biological replicates. **b.** Growth of cells hemizygous for the minor *PRIM1*rs2277339 allele (SNU-175PRIM1 G/–) expressing shGFP, positive control shPRIM1, or indicated putative allele-specific shRNAs targeting the G allele of *PRIM1*rs2277339 as measured by CellTiter-Glo luminescence, relative to day of assay plating. n = 3 biological replicates. One-tailed Student's t-test, *p = 0.03. **c.** Growth of heterozygous cells (SNU-175PRIM1 T/G) and cells hemizygous for the major *PRIM1*rs2277339 allele (SNU-175PRIM1 T/–) expressing shGFP, positive control shPRIM1, or indicated putative allele-specific shRNAs targeting the T allele of *PRIM1*rs2277339 as measured by CellTiter-Glo luminescence, relative to day of assay plating. n = 4 biological replicates. One-tailed Student's t-test, n.s. = not significant. **d.** Growth of heterozygous cells (SNU-175PRIM1 T/G) and cells hemizygous for the minor *PRIM1*rs2277339 allele (SNU-175PRIM1 G/–) expressing shGFP, positive control shPRIM1, or indicated putative allele-specific shRNAs targeting the G allele of *PRIM1*rs2277339 as measured by CellTiter-Glo luminescence, relative to day of assay plating. n = 3 biological replicates. One-tailed Student's t-test, n.s. = not significant. Data for panels a–d are presented as mean values +/- s.d. Source data provided as a Source Data file.

Supplementary Note 1

We verified that allele-specific inactivation of essential genes is possible in a heterozygous context. Gene-disrupting indels introduced by the error-prone non-homologous end joining (NHEJ) repair pathway make distinguishing the original genotype (S or R) of an edited allele challenging. Therefore, we compared the number of unaltered reads of each allele in heterozygous cells expressing either NA or AS sgRNA. If the sgRNA disrupts *PRIM1* in a non-allele specific fashion, we would expect a ratio of 1 between unaltered reads of each allele. We infected a Cas9-stable PRIM1$^{R/S}$ line with NA or AS sgRNA as described above and sequenced the target loci 4 and 18 days post-infection. As expected, we saw no substantial difference in the number of unaltered reads between the two alleles in PRIM1$^{R/S}$ cells expressing NA sgRNA (Supplementary Figure 2l). In contrast, PRIM1$^{R/S}$ cells expressing AS sgRNA showed significantly more unaltered reads from the resistant allele compared to the sensitive allele, with a ratio that increased over time ($p<0.0001$, Chi-square with Yates correction). We conclude that the AS sgRNA disrupts *PRIM1* in a SNP-specific manner even in a heterozygous context.

Supplementary Note 2

We confirmed that AS CRISPR disrupts *EXOSC8* in a SNP-dependent manner using deep sequencing. As with PRIM1, we assessed EXOSC8-targeting sgRNA efficiency at the early time point of four days post-infection. At this early time point, SW-579 (EXOSC8 R/R) and DV-90 (EXOSC8 S/S) cells infected with the EXOSC8 NA sgRNA showed approximately equal fractions of disrupted alleles (Figure 3c). However,

while DV-90 sensitive cells expressing AS sgRNA showed upwards of 90% disrupted alleles, SW-579 resistant cells under the same condition showed fewer than 3% disrupted alleles, a significant difference ($p<0.0001$, Chi-square with Yates correction). This result confirms that EXOSC8 AS sgRNA targets *EXOSC8* in a SNP-specific manner.

Supplementary Note 3

We verified that the specificity of the AS sgRNA for EXOSC8S cell lines was not due to a lack of Cas9 activity or EXOSC8 essentiality in the EXOSC8R cell lines. We transduced all four cell lines with a non-allele specific (NA) targeting sgRNA, which successfully ablated EXOSC8 expression in all contexts (Figure 3d). Cells expressing EXOSC8-targeting sgRNA showed significant decreases in proliferation relative to LacZ-targeting control ($p<0.01$ in all cases, one-tailed Student's t-test), confirming that this gene is cell-essential (Figure 3e, Supplementary Figure 4c-f).

Supplementary Discussion

As an enzyme, PRIM1 represents a potential target for small-molecule drug development. However, while several potential inhibitors of human DNA primase have been proposed, none have yet advanced to clinical stages of development[1]. Efforts to discover additional putative primase inhibitors may benefit from approaches used to target bacterial and viral primases. For instance, a combined fragment-based/virtual screening approach has been used to identify novel inhibitors of the T7 bacteriophage primase. Such an approach may aid PRIM1-targeting lead-compound discovery by

eliminating the challenging task of initial primase functional screening for large numbers of compounds[2].

The residue coded for by *EXOSC8*[rs117135638] lies on the interface of the *EXOSC8* gene product Rrp43 and exosome complex member Mtr3, raising the possibility of developing an allele-specific inhibitor of exosome complex formation. Pharmacologic inhibition of protein-protein binding has generally proven challenging due to the large, often flat, surfaces involved in protein complex formation[3]. However, disruption of several clinically relevant protein-protein interactions has been achieved previously, as illustrated by small-molecule or peptidomimetic inhibitors of p53-Mdm2 binding[4–6], Notch complex assembly[7], and herpesvirus DNA polymerase complex assembly[8]. As with *PRIM1*, any such approach to targeting LOH of *EXOSC8* must also overcome the substantial hurdle of achieving allele specificity.

Supplementary References

1. Boudet, J., Devillier, J. C., Allain, F. H. T. & Lipps, G. Structures to complement the archaeo-eukaryotic primases catalytic cycle description: What's next? *Comput. Struct. Biotechnol. J.* **13**, 339–351 (2015).
2. Ilic, S. *et al.* Identification of DNA primase inhibitors via a combined fragment-based and virtual screening. *Sci. Rep.* **6**, 1–10 (2016).
3. Bakail, M. & Ochsenbein, F. Targeting protein-protein interactions, a wide open field for drug design. *Comptes Rendus Chim.* **19**, 19–27 (2016).
4. Lao, B. B. *et al.* Rational design of topographical helix mimics as potent inhibitors of protein-protein interactions. *J. Am. Chem. Soc.* **136**, 7877–7888 (2014).

5. Vassilev, L. T. *et al.* In Vivo Activation of the p53 Pathway by Small-Molecule Antagonists of MDM2. *Science (80-.).* **303**, 844–848 (2004).

6. Fasan, R. *et al.* Using a β-hairpin to mimic an α-helix: Cyclic peptidomimetic inhibitors of the p53-HDM2 protein-protein interaction. *Angew. Chemie - Int. Ed.* **43**, 2109–2112 (2004).

7. Moellering, R. E. *et al.* Direct inhibition of the NOTCH transcription factor complex. *Nature* **462**, 182–188 (2009).

8. Loregian, A., Marsden, H. S. & Palu, G. Protein-protein interactions as targets for antiviral chemotherapy. *Rev. Med. Virol.* **12**, 239–262 (2002).
