# Supplementary material for: Loss of heterozygosity of essential genes represents a widespread class of potential cancer vulnerabilities
Source: Nat Commun. 2020 May 20;11:2517. doi: 10.1038/s41467-020-16399-y (PMC7239950; doi:10.1038/s41467-020-16399-y)
Supplement: Supplementary file 2 — Description of Additional Supplementary Files [file 41467_2020_16399_MOESM2_ESM.pdf]

Description of Additional Supplementary Files

Supplementary Data 1: Cell-essential genes.

Supplementary Data 2: DAVID Biological Process GO Term enrichment analysis for cell-essential genes.

Supplementary Data 3: GEMINI vulnerabilities.

Supplementary Data 4: DAVID Biological Process GO Term enrichment analysis for GEMINI genes.

Supplementary Data 5: Annotation of protein-altering GEMINI vulnerabilities.
