# Supplementary material for: Loss of heterozygosity of essential genes represents a widespread class of potential cancer vulnerabilities
Source: Nat Commun. 2020 May 20;11:2517. doi: 10.1038/s41467-020-16399-y (PMC7239950; doi:10.1038/s41467-020-16399-y)
Supplement: Supplementary file 8 — Reporting Summary [file 41467_2020_16399_MOESM8_ESM.pdf]

nature research

Corresponding author(s): Caitlin Nichols, Brenton Paolella, Rameen Beroukhim

Last updated by author(s): Jan 31, 2019

Reporting Summary

Nature Research wishes to improve the reproducibility of the work that we publish. This form provides structure for consistency and transparency in reporting. For further information on Nature Research policies, see Authors & Referees and the Editorial Policy Checklist.

Statistics

For all statistical analyses, confirm that the following items are present in the figure legend, table legend, main text, or Methods section.

n/a	Confirmed	
☐	☒	The exact sample size (n) for each experimental group/condition, given as a discrete number and unit of measurement
☐	☒	A statement on whether measurements were taken from distinct samples or whether the same sample was measured repeatedly
☐	☒	The statistical test(s) used AND whether they are one- or two-sided<br>*Only common tests should be described solely by name; describe more complex techniques in the Methods section.*
☒	☐	A description of all covariates tested
☐	☒	A description of any assumptions or corrections, such as tests of normality and adjustment for multiple comparisons
☐	☒	A full description of the statistical parameters including central tendency (e.g. means) or other basic estimates (e.g. regression coefficient) AND variation (e.g. standard deviation) or associated estimates of uncertainty (e.g. confidence intervals)
☐	☒	For null hypothesis testing, the test statistic (e.g. F, t, r) with confidence intervals, effect sizes, degrees of freedom and P value noted<br>*Give P values as exact values whenever suitable.*
☒	☐	For Bayesian analysis, information on the choice of priors and Markov chain Monte Carlo settings
☒	☐	For hierarchical and complex designs, identification of the appropriate level for tests and full reporting of outcomes
☒	☐	Estimates of effect sizes (e.g. Cohen's d, Pearson's r), indicating how they were calculated

Our web collection on statistics for biologists contains articles on many of the points above.

Software and code

Policy information about availability of computer code

Data collection	Immunoblot images were collected using ImageQuant LAS 4000 (Version 1.3). qRT-PCR data were collected using ABS 7300 System SDS Software (Version 1.4.0.27). Protein lysate concentration data were collected using Microplate Manager Macintosh OS X (MPMIII Version 1.0d1). Cell-Titer Glo luminescence data were collected using BMG Labtech Optima (Version 2.20R2).
Data analysis	Data were analyzed using R Studio (Version 1.1.456), R (3.5.1), and Prism 7. Sanger sequencing data were analyzed using SnapGene Viewer (4.2.11).

For manuscripts utilizing custom algorithms or software that are central to the research but not yet described in published literature, software must be made available to editors/reviewers. We strongly encourage code deposition in a community repository (e.g. GitHub). See the Nature Research guidelines for submitting code & software for further information.

Data

Policy information about availability of data

All manuscripts must include a data availability statement. This statement should provide the following information, where applicable:

- Accession codes, unique identifiers, or web links for publicly available datasets
- A list of figures that have associated raw data
- A description of any restrictions on data availability

We include a data availability statement in the manuscript.

Field-specific reporting

Please select the one below that is the best fit for your research. If you are not sure, read the appropriate sections before making your selection.

☒ Life sciences ☐ Behavioural & social sciences ☐ Ecological, evolutionary & environmental sciences

For a reference copy of the document with all sections, see nature.com/documents/nr-reporting-summary-flat.pdf

Life sciences study design

All studies must disclose on these points even when the disclosure is negative.

Sample size	No sample size calculation was performed—growth assays were plated at five technical replicates per sample and RT-PCR assays at three technical replicates per sample to capture relevant technical variation.
Data exclusions	Individual qRT-PCR or CellTiter-Glo wells were excluded from analyses if they differed dramatically from the other two or four technical replicates.
Replication	All growth assays were repeated with at least two or three biological replicates (see Figures 2 and 3, Supplementary Figures 2 and 4). All attempts at validation were successful.
Randomization	Not relevant to this study—samples for cellular assays were plated from the same culture on the same day at the same time.
Blinding	Investigators were not blinded—needed to maintain cell line and sgRNA labels for tracking and analysis of data.

Reporting for specific materials, systems and methods

We require information from authors about some types of materials, experimental systems and methods used in many studies. Here, indicate whether each material, system or method listed is relevant to your study. If you are not sure if a list item applies to your research, read the appropriate section before selecting a response.

Materials & experimental systems

n/a	Involved in the study	
☐	☒	Antibodies
☐	☒	Eukaryotic cell lines
☒	☐	Palaeontology
☒	☐	Animals and other organisms
☒	☐	Human research participants
☒	☐	Clinical data

Methods

n/a	Involved in the study	
☒	☐	ChIP-seq
☒	☐	Flow cytometry
☒	☐	MRI-based neuroimaging

Antibodies

Antibodies used	EXOSC8—Proteintech #11979-1-AP, Lot #00014147<br>PRIM1—Cell Signaling Technology #4725, Ref: 07/2016, Lot #1<br>Vinculin—Sigma #V9131, Clone hVIN-1; Batch #036M4797V, #118M4777V, #018M4779V
Validation	EXOSC8: validated in 4 immunoblots on manufacturer's website and two publications (Pubmed IDs: 24989451, 23844004); signal decreases dramatically in lines with targeted EXOSC8 inactivation by two different sgRNAs.<br>PRIM1: validated in 1 immunoblot on manufacturer's website; signal decreases dramatically in lines with targeted PRIM1 inactivation by two different sgRNAs.<br>Vinculin: validated in 1 immunoblot on manufacturer's website and numerous publications.

Eukaryotic cell lines

Policy information about cell lines

Cell line source(s)	Cell lines were originally purchased from ATCC or the Broad Institute of MIT and Harvard.
Authentication	Cell lines were genotyped for the SNP of interest using Sanger and/or next-generation sequencing.
Mycoplasma contamination	Cell lines were not tested for mycoplasma contamination.
Commonly misidentified lines<br>(See ICLAC register)	No commonly misidentified cell lines were included in this study.
